# Nonparametric Determination of the Committor in Multimolecular Systems

**DOI:** 10.1021/acs.jctc.5c01427

**Published:** 2025-10-19

**Authors:** Lair F. Trugilho, Stefan Auer, Leandro G. Rizzi, Sergei V. Krivov

**Affiliations:** † Faculty of Biological Sciences, 4468 University of Leeds, Leeds LS2 9JT, United Kingdom; ‡ Departamento de Física, Universidade Federal de Viçosa (UFV), Av. P. H. Rolfs s/n, 36570-900 Viçosa, Brazil; § School of Chemistry, University of Leeds, Leeds LS2 9JT, United Kingdom; ∥ Astbury Center for Structutral Molecular Biology, University of Leeds, Leeds LS2 9JT, United Kingdom

## Abstract

A fundamental problem in analyzing large longitudinal data sets modeling dynamics in multimolecular systems is determining the underlying free-energy landscapes as a function of the committor, the optimal reaction coordinate. Here, we demonstrate that by combining a nonparametric approach with a systematic method for generating permutationally invariant collective variables, the committor can be effectively determined to describe multimolecular aggregation in a system with anisotropic interactions. The optimality of the committor is verified by a stringent validation test, and it is shown that the diffusive model along the committor yields kinetic properties identical to those derived from the original dynamics. Our method is general and relevant to the large machine learning community developing methods to determine the committor from longitudinal data sets.

## Graphical abstract

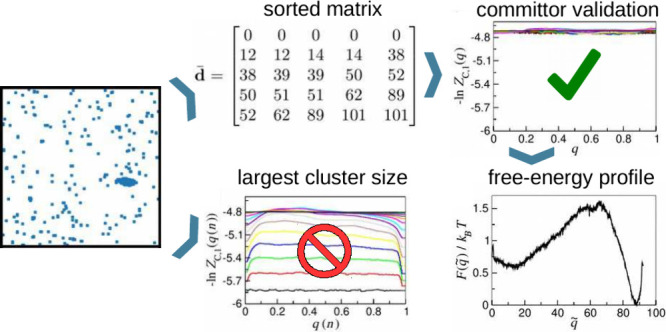

Multimolecular systems undergoing transitions between states with distinct physical properties are ubiquitous in nature, and prominent examples include condensation of small molecules,^1^ protein crystallization,^2^ and macromolecular aggregation.^3–5^ The steady-state kinetics of transitions in those systems is usually described by projecting the very-high dimensional configurational space of the molecules onto a few reaction coordinates (RCs). The remaining large number of degrees of freedom are modeled as noise by a stochastic model, such as the diffusive models described by the Smoluchowski and Fokker–Planck-like equations.^6,7^ The RC choice, however, is one of the pivotal difficulties that may lead to discrepancies when comparing simulated transition rates with their experimentally measured counterparts.^8^

In the case of physically motivated RCs,^9–11^ the projection usually introduces memory effects, and because of their non-Markovian character, there is no guarantee that the corresponding diffusive models can be used to describe quantitatively the system’s dynamics at arbitrary time scales.^12,13^ Alternatively, the formalism of optimal reaction coordinates^14^ selects RCs that minimize non-Markovian effects so that dynamics can be accurately described as simple diffusion on the corresponding free-energy landscape. For equilibrium stochastic dynamics between two boundary states, the committor function is considered an optimal RC.^15,16^ It is defined as the probability for the trajectory to reach one boundary state (*B*) before reaching the other one (*A*) starting from any given configuration.^17^ It has been shown that, despite the dynamics projected on the committor coordinate *q* being not strictly Markovian, a diffusive model along it can be used to exactly compute the equilibrium flux *J* = *D*(*q*)*p*_eq_(*q*),^15,18^ the mean transition path times (MTPT) τ̂ ,^14^ and the mean first passage times (MFPT)^15,19,20^

1 $$\tau_{B\to A}=\int_{0}^{1}\frac{dq^{\prime }}{p_{\mathrm{eq}}\left(q^{\prime }\right)D\left(q^{\prime }\right)}\int_{q^{\prime }}^{1}p_{\mathrm{eq}}\left(q^{\prime \prime }\right)dq^{\prime \prime }$$

between any two points on the committor landscape. These results hold for arbitrarily complex equilibrium stochastic dynamics in configuration space and do not require a separation of time scales. Here *D*(*q*) and *p*_eq_(*q*) are the temperature-dependent diffusion coefficient and equilibrium probability density function, respectively. While the description of the system’s dynamics using the committor is rather simple since it only depends on the free-energy profile *F*(*q*)/*k*_*B*_ *T* ∝ −ln *p*_eq_(*q*), as well as the diffusion coefficient *D*(*q*), its determination is an unsolved problem for multimolecular systems. In principle, the committor can be computed from the multidimensional Smoluchowski-like equation for diffusing particles,^14,21^ but due to its complexity, it has only been solved for low dimensional systems.^22^ In this letter, we consider the high-dimensional case of multimolecular aggregation, and present a systematic way of constructing permutationally invariant collective variables (CVs), which can be used in nonparametric optimization to determine the committor. The optimization scheme has been validated previously in unimolecular systems, such as the analysis of large scale atomistic protein folding simulations.^23,24^

The presented approach is timely, given the rapid development of computer hardware, which has led to an explosion of large, high-dimensional longitudinal data sets modeling transitions in multimolecular systems. These data sets require sophisticated analysis and interpretation. Recently, numerous machine learning approaches have been proposed to determine the committor, but they remain limited to relatively simple or low-dimensional systems.^25–27^ Other relevant developments include path sampling which uses machine learned committor estimates to improve sampling and to iteratively refine it.^28^ Additionally, the committor-based bias potential introduced in ref 29 iteratively improves sampling of transition states leading to better committor representations. Independent of these efforts for constructing optimal RCs to describe transitions in multimolecular systems, there is a lack of well-established benchmark systems, which are easy to simulate yet challenging to analyze. Recent studies either considered complex systems with relatively simple RCs (e.g., ion association^28^), or simple model systems (e.g., alanine dipeptide^26,27,29^ or Mueller potential^25–27,29^ ). Here, we considered a system with a low simulation cost, but rather complex RC landscape. In this way, it can serve as one of such benchmark systems, and the described approach together with the stringent optimality criterion, provides state-of-the-art results for aggregating phenomena. As it is feasible to sufficiently sample this system with long equilibrium simulations, scarce sample problems addressed in refs 26–29 are not critical. Nevertheless, our methodology can be combined with nonequilibrium sampling using the nonequilibrium nonparametric approach of ref 30.

Here, we consider stochastic dynamics in a multidimensional configuration space X⃗ , where we are interested in describing the dynamics of a reaction between two specified boundary states, *A* and *B*. To achieve this, we introduce a scalar RC, r(X⃗), which projects X⃗ onto a one-dimensional coordinate such that r(X⃗∈A)=0 and r(X⃗∈B)=1 . In general, the dynamics projected onto this coordinate is non-Markovian due to the loss of information associated with the dimensionality reduction.^12,13^ This non-Markovian behavior can be accurately described using the generalized Langevin equation with a memory kernel, which can be derived via the Mori-Zwanzig formalism.^31^ However, computing the memory kernel for practical cases is often challenging. Neglecting memory effects, dynamics can be approximated using a simple diffusive model, where the free energy *F*(*r*) and diffusion coefficient *D*(*r*) are computed as functions of the RC from its time series r(kΔt)=r(X⃗(kΔt)) sampled with Δ*t* (e.g., simulation step). Such models generally predict faster kinetics manifested as shorter MFPT, higher flux, and lower apparent free energy barriers.^23,32–35^

To improve the accuracy of this description, one can optimize the RC to mitigate these non-Markovian effects by employing variational approaches.^33–38^ Consider, specifically, the nonparametric approach of RC optimization,^23,24,30,39^ which improves RC iteratively. It considers iterative variations of the putative RC time series, $r_{m+1}\left(\mathrm{X⃗}\left(k\Delta t\right)\right)=r_{m}\left(\mathrm{X⃗}\left(k\Delta t\right)\right)+\delta r\left(\mathrm{X⃗}\left(k\Delta t\right)\right)$ , where δr(X⃗(kΔt)) satisfies δr(X⃗(kΔt)∈A)=δr(X⃗(kΔt)∈B)=0 to preserve boundary conditions. For optimizing the committor, we used the variation

2 $$\delta r\left(r_{m},x_{m}\right)=\overset{l}{\underset{i=0}{\sum }}\overset{l-i}{\underset{j=0}{\sum }}a_{ij}{\left(r_{m}\right)}^{i}{\left(x_{m}\right)}^{j}$$

where $x_{m}=x_{m}\left(\mathrm{X⃗}\left(k\Delta t\right)\right)$ denotes a randomly chosen CV time series in the *m*-th iteration which will be described later. This definition is convenient as the optimal variational coefficients *a*_*ij*_ can be found analytically by minimizing the total squared displacement (TSD) functional,^30,39^ see also the Supporting Information (SI). We note that as the approach deals directly with the CV time series, there is no need to compute the full RC function r(X⃗) , in this sense it is nonparametric, as opposed to most machine learning approaches, which try to parametrize the function r(X⃗) with, e.g., an artificial neural network. The convergence of such an optimization scheme can then be verified through the *Z*_*C*,1_ validation criterion for the committor, which states that if the putative RC *r* closely approximates the committor *q*, function *Z*_*C*,1_(*r*, Δ*t*) is constant, i.e., independent of *r* and Δ*t*, and equal to the number of transitions *N*_*AB*_ between the specified boundary states.^15^ *Z*_*C*,1_(*r*, Δ*t*) can be straightforwardly computed from the RC time-series *r*(*k*Δ*t*) as $\sum_{k}^{\prime }|r(k\Delta t+\Delta t)-r(k\Delta t)|$ , where the prime indicates that the sum is over all transitions such that *r* is between *r*(*k*Δ*t* + Δ*t*) and *r*(*k*Δ*t*)^15^ (further details in the SI).

It is worth noting that, assuming diffusive dynamics over a putative RC *r*, the diffusion coefficient can be accurately estimated as^15^

3 $$D\left(r,\Delta t\right)=\frac{Z_{C,1}\left(r,\Delta t\right)}{\Delta tZ_{H}\left(r,\Delta t\right)}$$

where the histogram *Z*_*H*_(*r*, Δ*t*) is related to the equilibrium distribution *p*_eq_(*r*) ∝ *Z*_*H*_(*r*, Δ*t*) and free-energy profile *F*(*r*)/*k*_*B*_ *T* ∝ −ln *Z*_*H*_(*r*, Δ*t*). In this way, *F*(*r*)/*k*_B_ *T* and *Z*_*C*,1_(*r*, Δ*t*) completely determine the diffusive models over the chosen RC, whether it displays Markovian dynamics or not.^40^ In particular, for the optimal coordinate *q*, the criteria that *Z*_*C*,1_(*q*, Δ*t*) is independent of Δ*t* ensures that the diffusion coefficient *D*(*q*) given by eq 3 is also independent of Δ*t* (this is because the non-normalized histogram scales as *Z*_*H*_(*q*, Δ*t*) ∼ Δ*t* ^–1^), just like it is for normal (i.e., Markovian) diffusion.

What remains is the task of identifying a suitable set of CVs {*x*_*m*_} for a multimolecular system with *N* molecules. In particular, such CVs need to respect the system’s symmetries, which for multimolecular systems are invariance under translations, rotations and permutation of identical molecules.^41^ For the first two, one can just consider distances *d*_*ij*_ between molecules noting that, if periodic boundary conditions are used in simulations, the minimum image convention should be used. Permutational invariance has proven more difficult to ensure and here we propose a systematic way of constructing such invariant CVs: We take the full matrix of distances **d** with *N* × *N d*_*ij*_ elements and sort it (from small to large) sequentially along its two axis, i.e., first inside each column and then inside each row. In this way, the time series of the elements ${\mathrm{d̅}}_{ij}={\mathrm{d̅}}_{ij}\left(k\Delta t\right)$ of the sorted matrix d̅ compose the set of invariant CVs that can be used as *x*_*m*_ in eq 2 (see the SI for details).

To demonstrate the applicability of our approach for multimolecular aggregation, we consider a lattice system to compute its committor *q* along with the diffusive model, i.e., the free-energy profile *F*(*q*)/*k*_*B*_ *T* and diffusion coefficient *D*(*q*). As described in refs 40, 42 , the system is defined as a regular two-dimensional square lattice of size *L* = 200 containing *N* = 400 molecules. Besides its position, each molecule has an additional degree of freedom that determines its orientation, which can be of two types. The interaction between neighbor molecules is then defined depending on their relative orientation, which can be stronger, ψ_*s*_, for aligned molecules or weaker, ψ_*w*_, for nonaligned ones. In this way, the quantity ξ = ψ_*s*_/ψ_*w*_ is a measure of the interaction anisotropy. Similarly to ref 40 we perform Metropolis Monte Carlo (MC) simulations in the canonical (*NVT*) ensemble at a temperature *T* = T* where the system undergoes a first-order phase transition between diluted and aggregated states (in the SI we indicate the value of T* for different ξ). For the data production runs we performed six long equilibrium MC simulations, where each simulation consists of 10^8^ MC steps (MCs), and configurations were saved at every Δ*t*_0_ = 400MCs.

First, for the sake of comparison, we show results obtained using the number of molecules in the largest aggregate *n* as RC, which is commonly used in the context of the classical nucleation theory.^43^ We computed the committor as a function of *n*, *q*(*n*), to evaluate the optimality of *n*. To do so, we performed the optimization procedure given by eq 2 with *x*_*m*_ = *n*(*k*Δ*t*) for all iteration steps *m* until convergence is reached (*m* = 20). As the transformation *n* → *q*(*n*) does not change the RC optimality, the profile along *q*(*n*) is representative of the original *n* RC.^15^ Moreover, using the *q*(*n*) coordinate one can directly apply the committor validation test to see whether *q*(*n*) approaches the true multidimensional committor function *q*. As can be seen in Figure 1 (a), −ln *Z*_*C*,1_(*q*(*n*), Δ*t*) increases from −5.8 to −4.8 as Δ*t* increases from Δ*t*_0_ to 2^15^Δ*t*_0_, indicating that *q*(*n*) failed to pass the committor validation test. For the *q*(*n*) coordinate the boundary states *A* and *B* are defined by the corresponding minima on the free-energy profile *F*(*n*)/*k*_*B*_ *T*.

**Figure 1:**
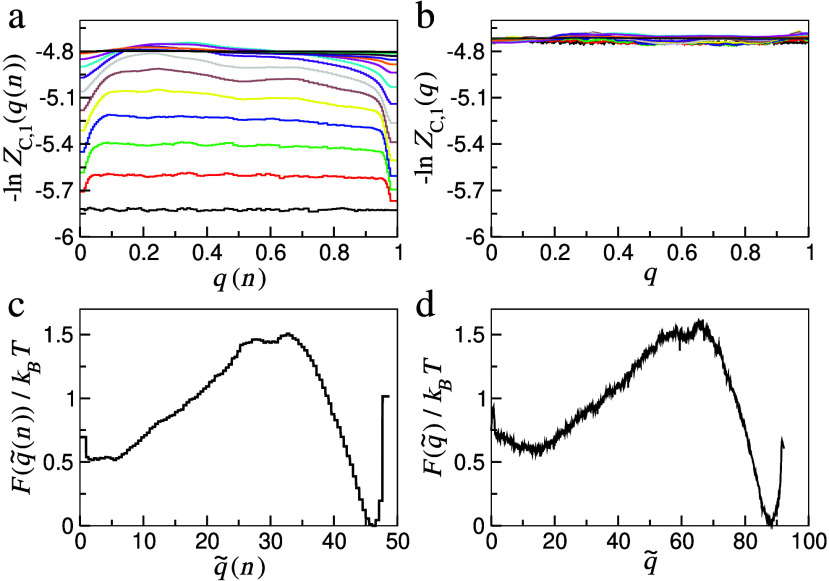
Functions −ln *Z*_*C*,1_(*r*, Δ*t*) and free-energy profiles *F*(*r*)/*k*_*B*_ *T* for the *n*-based RC *q*(*n*) and the optimized committor *q*. Panels (a) and (b) show the *Z*_*C*,1_-based committor validation tests for both RCs, where different colors correspond to different Δ*t* = 2^*i*^Δ*t*_0_, with *i* = 0, 1, 2, ..., 15 (from the bottom to the top). In panels (c) and (d), the profiles are presented as a function of the rescaled RCs where *D*(*r*, Δ*t*_0_) = 1.

Now we turn our attention to the putative committor RC obtained from the optimization procedure (eq 2) using {*x*_*m*_} as the CVs ( ${\mathrm{d̅}}_{ij}$ ) that respects all the symmetries of the system, for details see the SI. It can be seen in Figure 1 (b) that the constructed committor satisfactorily passed the validation test since *Z*_*C*,1_(*q*, Δ*t*) is constant, that is, it only fluctuates near the expected value −ln *Z*_*C*,1_(*q*, Δ*t*) ≈ −ln *N*_*AB*_ ≈ −4.72 for various Δ*t* up to statistical uncertainty, roughly estimated as $1/\sqrt{2N_{AB}}\approx 0.07$ . Here, the boundary states were defined in a more accurate, systematic way, by allowing optimization of the putative RC (soft committor) around the corresponding free-energy minima (see the SI for details). As the constructed optimal coordinate *q* passed the validation test, eq 3 implies that the diffusion coefficient *D*(*q*, Δ*t*) is independent of Δ*t*. On the other hand, for the suboptimal coordinate *q*(*n*), our estimates for *Z*_*C*,1_(*q*(*n*), Δ*t*), and hence for the diffusion coefficient *D*(*q*(*n*), Δ*t*) depend on sampling interval (observation time scale) Δ*t*, indicating the presence of strong non-Markovian effects. Interestingly, the non-Markovian behavior of *q*(*n*) and *n* is similar to the one observed when considering the energy as a RC.^40^

Next, we include in Figure 1(c) and (d) the free-energy profiles $F\left(\mathrm{q̃}\left(n\right)\right)/k_{B}T$ and $F\left(\mathrm{q̃}\right)/k_{B}T$ , where q̃(n) and q̃ are the rescaled coordinates where the diffusion coefficient is unitary,^32,44^ $D\left(\mathrm{q̃},\Delta t_{0}\right)=1$ and $D\left(\mathrm{q̃}\left(n\right),\Delta t_{0}\right)=1$ (see the SI for implementation details), so that the diffusive model and kinetics are specified by the free-energy profile alone. The comparison between the two free-energy profiles indicates that the free-energy barriers are almost the same, so the main difference is due to the ranges observed over q̃(n) and q̃ . While for q̃ the minima are separated by $\Delta \mathrm{q̃}\equiv {\mathrm{q̃}}_{B}-{\mathrm{q̃}}_{A}\approx 90$ , the *n*-based variable shows a separation around $\Delta \mathrm{q̃}\left(n\right)\equiv \mathrm{q̃}{\left(n\right)}_{B}-\mathrm{q̃}{\left(n\right)}_{A}\approx 45$ . As the diffusion coefficients over those coordinates are unitary, the smaller separation over q̃(n) implies a higher diffusivity for the *n*-based coordinate so that the related diffusive model leads to faster kinetics, with e.g., the MFPT τ_*B*→*A*_ = 1.8 × 10^3^Δ*t*_0_ calculated via eq 1 that is smaller than the τ_*B*→*A*_ = 4.7 × 10^3^Δ*t*_0_ determined directly from the time series *n*(*k*Δ*t*) (See Table 1). On the other hand, for the proposed optimal coordinate *q*, τ_*B*→*A*_ = 5.4 × 10^3^Δ*t*_0_ calculated via eq 1 is very close to the value τ_*B*→*A*_ = 5.3 × 10^3^Δ*t*_0_ extracted directly from the series *q*(*k*Δ*t*).

**Table 1:** Estimates for MFPT *τ*_*B*→*A*_ and Number of Transitions *N*_*AB*_ Obtained from Both *n*-Based RC *q*(*n*) and Commitor *q* for Systems with Different Anisotropies *ξ* ^a^

	ξ = 1	ξ = 3	ξ = 5	ξ = 7
τ_*B*→*A*_ from eq 1 with *q*(*n*)	1.8	3.3	3.6	6.2
τ_*B*→*A*_ from time series of *q*(*n*)	4.7	9.7	13	22
*N*_*AB*_ from *Z*_*C*,1_(*q*(*n*), Δ*t*_0_)	336	171	161	114
*N*_*AB*_ from time series of *q*(*n*)	122	55	40	28
τ_*B*→*A*_ from eq 1 with *q*	5.4	11	15	26
τ_*B*→*A*_ from time series of *q*	5.3	10	14	25
*N*_*AB*_ from *Z*_*C*,1_(*q*, Δ*t*_0_)	113	52	38	26
*N*_*AB*_ from time series of *q*	112	53	37	27

a All times are given in units of 10^3^ × Δ*t*_0_.

Next we include results for systems with different anisotropies ξ to show how the estimates for the kinetic properties evaluated through the diffusive model over the committor *q* compare with those obtained from the physically motivated RC *q*(*n*). In addition to the estimates for the MFPTs evaluated through eq 1 and from the time series of the RCs, we include in Table 1 the values of the number of transitions *N*_*AB*_ which can be computed not only directly from the times series but also from^15^ *N*_*AB*_ = *∫Z*_*C*,1_(*r*, Δ*t*_0_)­d*r*. The values displayed in Table 1 corroborate the fact that the diffusive model along *q*(*n*) gives MFPTs and number of transitions that would correspond to aggregation kinetics that is about three to four times faster than the corresponding values determined directly from the time series *n*(*k*Δ*t*). Conversely, the values of τ_*B*→*A*_ and *N*_*AB*_ obtained from the diffusive model along our constructed optimal coordinate *q* give essentially the same results measured directly from the trajectories *q*(*k*Δ*t*). In the SI we include additional results obtained for larger systems sizes, as well as for the reverse MFPT τ_*A*→*B*_ and MTPT τ̂ , which indicates that the agreement between the diffusive model along the proposed committor *q* and the discrepancies for the *q*(*n*) coordinate are observed for all cases.

To demonstrate the generality of our approach, we also present results for a more realistic three-dimensional Lennard-Jones system governed by overdamped Langevin dynamics, as detailed in the Supporting Information. The proposed method successfully computes the committor and associated kinetic properties.

Finally, in Figure 2 we show the free-energy profiles $F\left(\mathrm{q̃}\right)/k_{B}T$ obtained for systems with different anisotropies ξ along the rescaled commitor RC q̃ . Interestingly, just as for the energy-based profiles,^42^ the free-energy barriers display a nonmonotonic behavior with anisotropy. Even so, as the values included in Table 1 indicate, anisotropy slows down overall kinetics, as measured by τ_*B*→*A*_ and *N*_*AB*_. This is explained by the monotonic decrease of diffusivity with increasing ξ, that is, the range $\Delta \mathrm{q̃}={\mathrm{q̃}}_{B}-{\mathrm{q̃}}_{A}$ between the two basins in Figure 2 increase monotonically with ξ.

**Figure 2:**
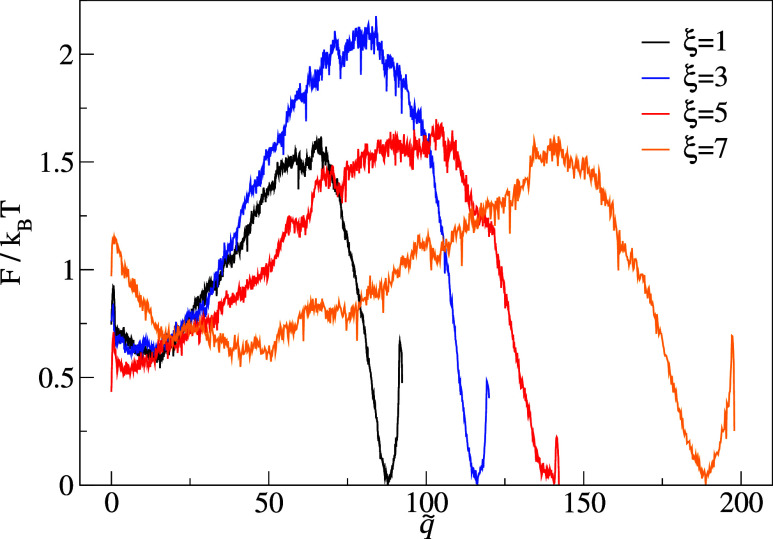
Free-energy landscapes $F\left(\mathrm{q̃}\right)/k_{B}T$ along the rescaled committor for different ξ.

To the best of our knowledge, the computation of committor *q* and its landscape for the entire aggregation process in finite yet large systems (i.e., with *N* ∼ 10^2^ molecules) is not available in the literature. Studies including committor analysis are generally restricted to the first steps of aggregation with only a handful of aggregating molecules (see e.g., refs 45, 46 where more sophisticated models of protein aggregation were considered). By considering a nonparametric variational approach^39^ supplied with a proposed set of permutationally invariant CVs, we confirmed that the diffusive model along the committor can be used to reproduce kinetic properties exactly. In addition, our results indicate that usual physically motivated RCs like *n* may not lead to accurate kinetic description at arbitrary time scales due to strong non-Markovian effects. When dealing with such suboptimal coordinates, more sophisticated stochastic models with a memory kernel are needed,^13^ in which case the free-energy profile loses its simple interpretation, or one needs to employ a suitable separation of time scales, as we did recently for the energy coordinate.^40^ The use of the committor as RC preserves the appealing simple picture of diffusion on the free-energy profile while avoiding the need for time scale separation. Such an accurate model of the dynamics can also be used to compute rigorously and in a direct manner, the pre-exponential factor,^23,34,47^ a key determinant of aggregation kinetics. Finally, it is worth mentioning that our approach is general and its implementation for the analysis of aggregation kinetics in more realistic multimolecular systems^1–5,45,46^ is straightforward. Our method is also of interest to the machine learning community, which is seeking methods to determine the committor for longitudinal data sets.^25–29^ In particular, the stringent *Z*_*C*,1_ validation test can be used to assess the accuracy of committor estimates obtained by various methods, including the many neural network architectures currently available, thereby greatly simplifying direct comparison between approaches.

## Supplementary Material



## Notes

It contains a practical example of how the permutationally invariant CVs are computed; additional methodological details for implementing the variational approach, defining boundary states, and computing kinetic quantities from diffusive models; additional results for larger lattice systems and a Lennard-Jones system; additional references (PDF)
