# Supplementary material for: Nonparametric Determination of the Committor in Multimolecular Systems
Source: J Chem Theory Comput. 2025 Oct 19;21(20):10080–5. doi: 10.1021/acs.jctc.5c01427 (PMC12573751; doi:10.1021/acs.jctc.5c01427)
Supplement: Supplementary file 1 [file ct5c01427_si_001.pdf]

Supporting Information (SI) for:

Nonparametric determination of the committor in multimolecular systems

Lair. F. Trugilho,[1,2] Stefan Auer,[3] Leandro. G. Rizzi,[2] Sergei. V. Krivov[1,4]

[1] Faculty of Biological Sciences, University of Leeds, Leeds LS2 9JT, United Kingdom
[2] Departamento de Física, Universidade Federal de Viçosa (UFV), Av. P. H. Rolfs s/n, 36570-900, Viçosa, Brazil
[3] School of Chemistry, University of Leeds, Leeds LS2 9JT, United Kingdom
[4] Astbury Center for Structutral Molecular Biology, University of Leeds, Leeds LS2 9JT, United Kingdom

Permutationally invariant CVs

The nonparametric approach described in the main text requires a set of CVs ($\{x_m\}$) invariant under the system's symmetries and that carry sufficient information about the system, e.g., they need to distinguish between aggregated and diluted states. In order to define such set of CVs, we worked with the Euclidean pairwise distances between molecules on the lattice. All distances are invariant under translations and rotations, but one needs further to define a set of distance-based time series that are invariant under exchange of indistinguishable molecules. This is usually done by either summing up all features (distances in our case) over all identical molecules (pairs of molecules for distances) or performing a permutationally invariant ordering [1]. We tried both of those approaches. For the former, we tried summing functions of the pairwise distances d_{ij}, such as exponential and Gaussian functions, with free parameters that varied, allowing for the construction of a set of CVs. For example, in Fig S1 (a) we consider the isotropic lattice system with $N = 400$, and show the $Z_{C,1}$ validation criteria for a putative committor RC obtained performing the optimization scheme with a set of CVs with $x_m = \sum_{i<j} \exp(-\alpha_m d_{ij})$, where $\{\alpha_m\}$ represent a set of four hundred logarithmically spaced numbers between 0.01 and 15. As can be seen in the figure, albeit been more optimal than n (compare with Fig. 1 of the main manuscript), the $Z_{C,1}$ functions still vary considerably with Δt when compared with the same plots for our final proposed committor, which we show again on Fig. S1 (d) at the same axis scale. Particularly, the computed $N_{AB} = 146$ from the diffusive model along the committor obtained with the sum-based CVs still deviates from the real value computed directly from the trajectories $N_{AB} = 112$. Other choices of functions gave similar results.

Regarding the sorting strategy, we started ordering all pairwise distances between the N molecules from the smallest to the highest as is usually done. The time series of each of those $N(N-1)/2$ distances is a permutationally invariant CV. But the large amount of such CVs led to difficulties when dealing with them: One can not use all of these time series together for optimization due to memory limitations. We tried using a representative fraction of those CVs. In Fig. S1 (b) we show the validation criteria for the resulting committor obtained from the optimization scheme with x_m consisting of the j_m-th smallest distances with $j_m = \text{int}(2^{8+i_m/50})$, $i_m = 0, 1, ..., 399$, where the function int() converts the argument to the nearest smallest integer. The results are very similar compared to the sum-based CVs, slightly better for $\Delta t = \Delta t_0$ (black curves). Both cases are worst even when compared with the committor obtained using only one axis of the sorted matrix (see below), of which we show the validation test on Fig. S1 (c). Not only the $Z_{C,1}$ functions fluctuates less in Fig S1 (c), but they are optimized in a more uniform way. The choice of the m-th distances out of $N(N-1)/2$ linearly spaced entries illustrate the difficulty of selecting the representative set of distances. Because of that, we implemented a different, more informative way of sorting those distances.

We considered the symmetric matrix $\mathbf{d}$ whose $N \times N$ elements are the distances between any two molecules i and j, d_{ij}. First, the two-dimensional matrix is sorted along one of its axis (see example below). Mathematically, the elements of the new matrix $\hat{\mathbf{d}}$, after sorting along the first index, have the property $0 = \hat{d}_{1j} < \hat{d}_{2j}, ..., < \hat{d}_{Nj}$ for all j ranging from 1 to N. Each molecule corresponds to specific index j and the related column contains the distance of the j-th labeled molecule to all others in a sorted way. As these columns are molecule dependent, none of the distances inside them are invariant. Even so, one can sort it again along the remaining index in order to get a completely sorted matrix $\bar{\mathbf{d}}$ where, in addition to the previous inequality, it also has $\bar{d}_{i1} < \bar{d}_{i2}, ..., < \bar{d}_{iN}$ for every i defining a row. Now, neither row i nor column j is molecule dependent and all the N^2 distances of the resulting matrix are invariant under permutations. So, by repeating this procedure for each distance matrix at each time step, the time series of each matrix element $\bar{d}_{ij}(k\Delta t)$ is an invariant CV. Although we have virtually the same huge number of CVs in this new way of sorting compared to the more common previous one, the new one turned out to produce more informative CVs and be more suitable for the optimization procedure. That is because one can work with one axis of $\bar{\mathbf{d}}$ at a time, avoiding memory issues, but then subsequently continue the optimization with other axes to improve the committor time series estimate. At the end, by using about ten axes with $m = 10000$ iteration steps for each, we were able to construct the putative optimized RCs which closely approach the committor for isotropic and anisotropic systems, as measured through the committor validation test (i.e., $Z_{C,1}$ is constant for

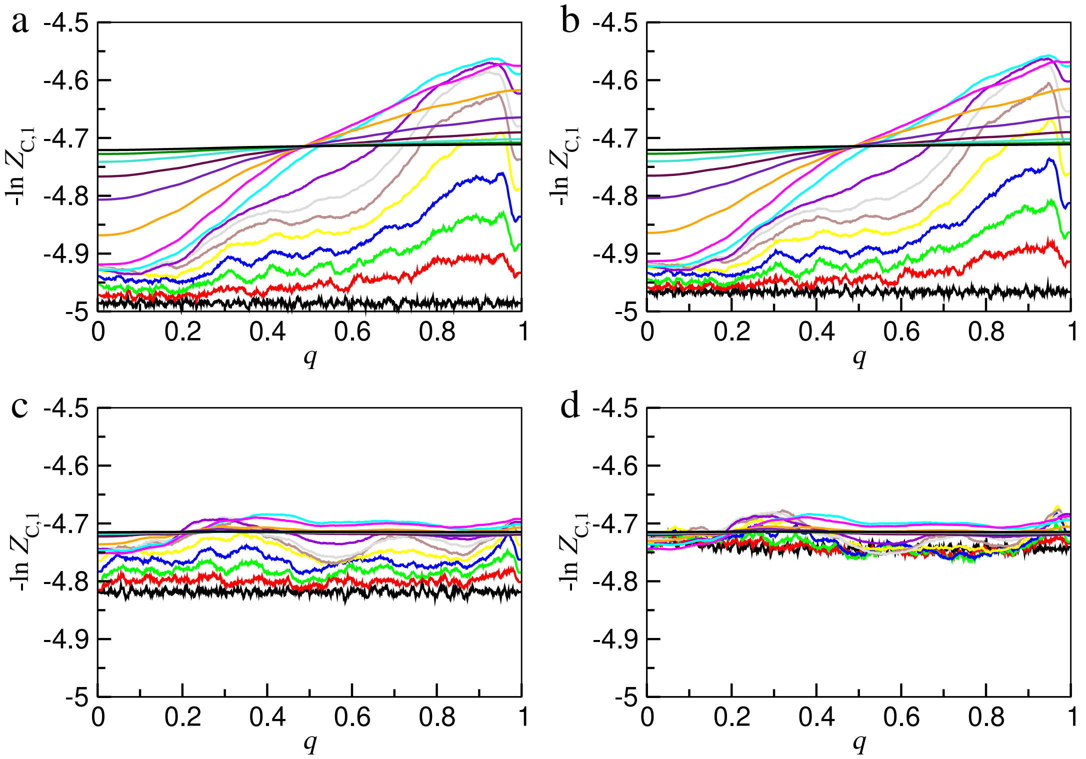

FIG. S1. Functions $-\ln Z_{C,1}(q, \Delta t)$ for different estimates of the committor q. (a) Optimization performed with $\{x_m\}$ obtained summing up exponential functions of the pairwise distances. (b) $\{x_m\}$ consisting of selected sorted distances in the usual way. (c) $\{x_m\}$ consisting of the entries of only one axis $\bar{d}_{i,7}$ of the sorted matrix. (d) Final estimate obtained employing eight axis of the sorted matrix. In all plots different colors correspond to different $\Delta t = 2^i \Delta t_0$, with $i = 0, 1, 2, ..., 15$ (from the bottom to the top).

the committor). Moreover, this way of sorting turn out to be more instructive since it allows comparing axes to find which are the best ones. For example, we found that axes between $\bar{d}_{i,5}$ and $\bar{d}_{i,40}$ are the most effective for optimizing the committor coordinate, and axes much higher than that give worse results. This can be explained physically since much higher axes contain only the highest distances between molecules, which contain mostly information about diluted molecules, but not about the aggregated ones, so these CVs are not expected to distinguish well the diluted and aggregated states of the system. In Fig. S1 (c) we show the validation test for the putative RC obtained taking as $\{x_m\}$ only one axis $\bar{d}_{i,7}$ (a total of 400 CVs since $N = 400$) of the sorted matrix and the RC is already very close to the committor. Particularly, this putative RC is much more optimal than the ones obtained by more usual strategies described above considered in Fig S1 (a) and (b) with the same number of CVs (400). Moreover one can continue optimization by subsequently considering other axes in order to improve the putative RC. In Fig. S1 (d) we show the validation test considering eight axis uniformly distributed between $\bar{d}_{i,7}$ and $\bar{d}_{i,42}$ and the resulting RC approaches almost exactly, i.e., within statistical error, the true committor function. Importantly, simply increasing the number of CVs employed in the other two strategies described before does not seem to significantly improve the achieved results, since no substantial improvements were detected going from 65 to the reported 400 number of CVs. It seems that when dealing with the sorted distances in the usual way one would need to employ a clever way of selecting the representative subset of CVs, which seems to be much more simple using the sorted matrix that we employ.

To illustrate the procedure, let us consider a hypothetical system with only $N = 5$ molecules, with the symmetric matrix of distances between molecules $\mathbf{d}$ given by:

$$\mathbf{d} = \begin{bmatrix} 0 & 101 & 89 & 52 & 38 \\ 101 & 0 & 12 & 50 & 62 \\ 89 & 12 & 0 & 39 & 51 \\ 52 & 50 & 39 & 0 & 14 \\ 38 & 62 & 51 & 14 & 0 \end{bmatrix}. \tag{1}$$

Now, sorting inside each column one gets $\hat{\mathbf{d}}$:

$$\hat{\mathbf{d}} = \begin{bmatrix} 0 & 0 & 0 & 0 & 0 \\ 38 & 12 & 12 & 14 & 14 \\ 52 & 50 & 39 & 39 & 38 \\ 89 & 62 & 51 & 50 & 51 \\ 101 & 101 & 89 & 52 & 62 \end{bmatrix} . \tag{2}$$

Finally, sorting inside each row one gets the final completely sorted matrix $\bar{\mathbf{d}}$:

$$\bar{\mathbf{d}} = \begin{bmatrix} 0 & 0 & 0 & 0 & 0 \\ 12 & 12 & 14 & 14 & 38 \\ 38 & 39 & 39 & 50 & 52 \\ 50 & 51 & 51 & 62 & 89 \\ 52 & 62 & 89 & 101 & 101 \end{bmatrix} . \tag{3}$$

Taking fixed positions, say $\bar{d}_{2,5}$ here, and repeating the sorting procedure for every frame in the trajectory, one obtains $\bar{d}_{2,5}(k\Delta t)$, a permutatonally invariant CV time-series.

Nonparametric variational approach

As described in the main text, the nonparametric approach updates iteratively the putative RC time series, starting with the seed RC time-series $r_0 = r_0(k\Delta t) = r_0(\vec{X}(k\Delta t))$, satisfying the boundary conditions $r_0(\vec{X}(k\Delta t) \in A) = 0$ and $r_0(\vec{X}(k\Delta t) \in B) = 1$. At each iteration m the coordinate $r_m = r_m(k\Delta t)$ is updated using $r_{m+1} = r_m + \delta r(r_m, x_m)$ with $\delta r(r_m, x_m)$ being a low dimensional polynomial of l-th degree (we set $l = 6$ for all optimizations in this work) multiplied by a boundary function $\tilde{I}_b$, which ensures that variation is zero at boundaries,

$$\delta r(r_m, x_m) = \tilde{I}_b \sum_{i=0}^{l} \sum_{j=0}^{l-i} a_{ij} (r_m)^i (x_m)^j, \tag{4}$$

and $x_m = x_m(k\Delta t)$ corresponds to the randomly chosen invariant CV in the m-th step, e.g., the CVs extracted from the sorted matrix $\bar{\mathbf{d}}$. Here, a_{ij} are obtained invoking the variational principle where the total squared displacement (TSD) is minimum for the committor RC [2, 3], that is, at each iteration the parameters are evaluated from the minimization of the functional

$$\min_{\{a_{ij}\}} \left\{ \sum_k [r_{m+1}(k\Delta t + \Delta t) - r_{m+1}(k\Delta t)]^2 \right\}, \tag{5}$$

where k runs over all the time series. It can be shown that this variational principle is equivalent to the committor equation (Eq. 6 in the next Section) between any two states [2], A and B. Moreover, it is useful to modulate each term of the polynomial in Eq. 4 by a common envelope function in order to focus optimization near particular values of r, for example, we used sigmoidal functions $1/(1+e^{\pm(r_m - \hat{r}_m)/b})$ to focus optimization either for values $r_m \gtrsim \hat{r}_m$ or $r_m \lesssim \hat{r}_m$ at each iteration where $b = 0.01$ (used here) defines the scale. We implemented that by drawing randomly $\hat{r}_m$ as well as the sign in the sigmoid at every tenth optimization step. In this way one focuses near both basins of the free-energy, where optimization gets exponentially shrunk [4]. Finally, it is worth noting that the coefficients a_{ij} minimizing expression 5 can be evaluated analytically and the explicit equations can be found in Ref. [5].

Boundary conditions and soft committor

Here we discuss the matter of defining boundary states, e.g., aggregated A and diluted B mentioned in the main text. Such boundaries are precisely defined within the committor mathematical definition. In the Markov State Model formalism, the committor probability can be computed by solving the system of linear equations [2]:

$$q_i = \sum_j P_{ji} q_j \tag{6}$$

where q_i is committor probability for state i, i and j denote states of convenient discretized configuration space and P_{ji} is the transition probability from state i to j. Formally, we have $P_{ji} = n_{ji}/n_i$ with n_i the equilibrium number of times state i is visited and n_{ji} the number of transitions from i to j. Those equations are to be solved under the boundary conditions $q_A = 0$ and $q_B = 1$. This is exemplified at the top of Fig. S2, where we show a simple example with a few nodes. Each point in that figure corresponds to a node i and the system can move through those nodes with probabilities defined by P_{ij} represented by the connections between the points. The committor between

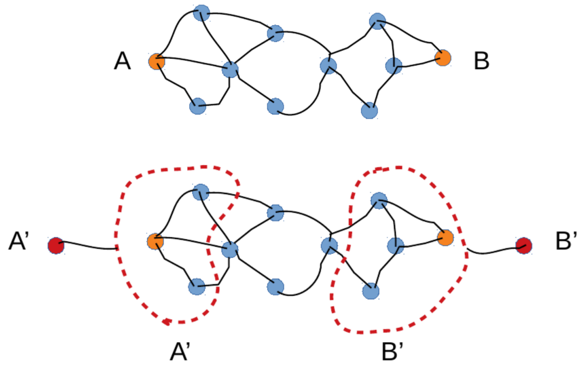

FIG. S2. Illustration of the “rigid” (top) and “soft” (bottom) boundary conditions defining the committor function. Points represent each node, while lines represent their connections. At the top, boundaries A and B are defined within the original network. At the bottom, all nodes in regions A' and B' near each basin, i.e. inside the dotted contours, are connected to artificial nodes which will serve as the boundary.

any two states A and B can be calculated by solving Eq. 6 with the appropriate boundaries. Alternatively, such boundary states are also well-defined in the Smoluchowski equation formalism, where the committor is the solution of a partial differential equation under analogous boundary conditions (see e.g., Refs. [6, 7]).

Although precisely defined, the choice of boundaries is still arbitrary, since we can choose any two nodes for it. The exact values are chosen depending on the problem at hand. Usually, one is interested in transitions between states that present different macroscopic properties, so it is desired to choose more distanced nodes in configuration space. For instance, in our example, one could choose the most distant nodes (the orange ones) to fix boundaries, as in the top of Fig S2. This choice of “rigid” boundary conditions corresponds to the usual committor function.

Although this is in principle a quite simple procedure, it can be difficult to determine such boundaries in multidimensional systems. In practice, boundary states are usually defined in a “rigid-like” way, choosing a physically motivated order parameter (OP) O that separates the two states, e.g., aggregated and diluted in our case. The free-energy profile along the OP should present two minima at O_A and O_B and one can try to define boundaries near each one of those minima, in a way that, e.g., if $O < O_A$ the system is considered on the aggregated state and if $O > O_B$ it is considered on the diluted state. In this way, all states with $O < O_A$ are merged in state A represented by node q_A and similarly for state B. We tried such a scheme for our aggregating system, using both (potential) energy E and the number of molecules in the biggest aggregate n as OPs. Although the free energy profiles along both order parameters (OPs) clearly exhibit two minima, the defined boundary states depend on the specific OP chosen. Notably, the total number of transitions observed between the aggregated and diluted states, N_{AB}, computed form the trajectory, varies significantly across different OPs, e.g., E or n here. This indicates that each OP define different sets of states.

As an alternative, one can define “soft” boundary conditions. This can be done by delimiting regions of configuration space that are just roughly near each boundary. Then, those entire regions, which we call A' and B', are connected with additional nodes A' and B' outside the original network, but that will now serve as the boundary states, as illustrated in the bottom of Fig. S2. Differently from the “rigid” boundary conditions, the nodes inside regions A' and B' are not merged together to form unique nodes as they do not define the boundaries themselves and movements between the nodes inside these regions are still considered. Each node (i) inside A' and B' regions is connected to the artificial boundaries A' and B' defining $n_{A'i} = \lambda_A$ and $n_{B'i} = \lambda_B$, respectively. Thus, for the extended network, every time the system enters in regions A' and B' there are “effective” transitions to the artificial boundary nodes A' and B' determined by the free parameters λ_A and λ_B. Solving Eq. 6 for the extended network, we get what we call the soft committor function. The aim of this procedure is to determine an improved OP (soft committor), which stratifies nodes based on their closeness to the boundaries, and can be used to determine boundary conditions in a more robust manner. Then, boundaries are defined in a usual “rigid-like” way, near the minima of the soft committor OP. Regions A' and B' can be defined using any conventional CV that only weakly separates the two boundary states. Here, for aggregated and diluted state, one can use either E or n.

Soft committor can be computed using a similar iterative variational principle as that described in the previous Section. The only difference is that now a_{ij} are obtained from the minimization of the TSD over the extended

network, which reads

$$\begin{aligned}\min_{\{a_{ij}\}}\Bigg\{ &\sum_k \Big\{ [r_{m+1}(k\Delta t + \Delta t) - r_{m+1}(k\Delta t)]^2 \\ &+ 2I_{A'}(k\Delta t)\lambda_A[r_{m+1}(k\Delta t)]^2 \\ &+ 2I_{B'}(k\Delta t)\lambda_B[r_{m+1}(k\Delta t) - 1]^2 \Big\}\Bigg\}\end{aligned} \tag{7}$$

where k runs over all the time series, but has two additional terms related to the artificial movements between nodes inside regions A'/B' and the respective artificial nodes now defining the boundaries. $I_{A'}$ and $I_{B'}$ are indicator functions defined as

$$I_{A'}(k\Delta t) = \begin{cases} 1, & O(k\Delta t) < O_{A'} \\ 0, & O(k\Delta t) > O_{A'} \end{cases} \tag{8}$$

and analogously for $I_{B'}$. In this way, when summing up over all trajectory in Eq. 7, these two additional terms count the number of times the system enters regions A' and B', which multiplied by λ_A and λ_B, respectively, results in the number of artificial movements to the boundaries. The squared displacement related to those movements is just $[r_{m+1}(k\Delta t) - 0]^2$ for movements to boundary A' ($q_{A'} = 0$) and $[r_{m+1}(k\Delta t) - 1]^2$ for movements to boundary B' ($q_{B'} = 1$). The factor of two accounts for the fact that we consider movements in both directions $n_{iA'} = n_{A'i}$ (the detailed balance) and similar for boundary B.

Soft committor time series were constructed via the variational principle 7 supplied with the permutationally invariant CVs. We used different choices of CVs (E, n and even some of the invariant CVs) to determine the regions A' and B' and the resulting soft committor OPs gave a similar number of measured transitions between aggregated and diluted states. This indicates the robustness of the optimized OPs compared with the physically motivated OPs, from which we measure different numbers of transitions. In fact, it turned out that the resulting number of transitions obtained using soft committor as OP is closer to that obtained using directly n as OP, compared to E as OP. This means that n seems to be better OP than E, i.e., it separates better the boundary states. The soft committor OP is likely to separate better the boundary states because it takes into account the multidimensional dynamics of the system when optimizing Eq. 7 with the permutationally invariant CVs. The energy projection, for example, gives almost twice the number of transitions for the isotropic system and even higher values for the anisotropic cases, which indicates the mixing of states that are more distant in configuration space. A similar soft committor construction was employed in Ref. [8] in the context of the Smoluchowski equation formalism, but as an approximation to the usual committor function, not with the aim of defining the boundaries as we introduce here.

Histogram-based and cut-based free-energy profiles

The usual histogram-based free-energy for a general reaction coordinate time series $r(k\Delta t)$ is computed as

$$F(r, \Delta t) = -k_B T \ln Z_H(r, \Delta t) \tag{9}$$

where its partition function is the density of points in each bin $[r, r + \Delta r]$:

$$Z_H(r, \Delta t) = M_r/\Delta r \tag{10}$$

where M_r is the number of time series points in the bin and Δr is the size of the bins. As $Z_H(r, \Delta t)$ is related to the equilibrium distribution of coordinate r, its dependence with sampling interval Δt is just trivially $Z_H \sim \Delta t^{-1}$ since it is non-normalized, so that increasing the sampling interval just reduces the number of trajectory points considered in its computation.

The cut-based free energy profile, $Z_{C,a}$, is computed by summing up the transitions through r weighted by the associated coordinate displacement to the power a [9]:

$$Z_{C,a}(r, \Delta t) = \frac{1}{2}\sum_k |r(k\Delta t + \Delta t) - r(k\Delta t)|^a \Theta[(r(k\Delta t) - r)(r - r(k\Delta t + \Delta t))], \tag{11}$$

where Θ is the Heaviside step function, which ensures that only transition where r is between $r(k\Delta t)$ and $r(k\Delta t + \Delta t)$ are taken into account. Of particular interest is $Z_{C,1}$ which can be related to the diffusion coefficient assuming diffusive dynamics via

$$Z_{C,1}(r, \Delta t) = \Delta t D(r, \Delta t) Z_H(r, \Delta t). \tag{12}$$

and is associated with a stringent committor validation test. We note that if the dynamics is truly Markovian over r, $D(r, \Delta t)$ is, in fact, independent of Δt, but we let the explicit temporal dependence on $D(r, \Delta t)$ since a relation

like Eq. 12 can be used to inspect whether the dynamics projected at an arbitrary RC r has strong non-Markovian effects: $D(r, \Delta t)$ computed from Eq. 12 varying with Δt indicate the presence of non-Markovian effects.

As $Z_{C,1}$ equals half the sum of times the trajectory passes through point r after successive time displacements Δt, weighted by the length of each coordinate displacement, it can be written more compactly as

$$Z_{C,1}(r, \Delta t) = \frac{1}{2} \sum_k{}' |r(k\Delta t + \Delta t) - r(k\Delta t)| \tag{13}$$

where the prime indicates that the sum is over all trajectory snapshots k such that r is between $r(k\Delta t)$ and $r(k\Delta t + \Delta t)$. If the RC satisfies the committor (q) equation (Eq. 6), then $Z_{C,1}(q, \Delta t)$ is independent of both coordinate q and Δt, except for the boundary nodes [2]. This issue can be simply rectified using the ensemble of transition path segments rather than the full trajectory for computing $Z_{C,1}$. These segments are constructed by partitioning the trajectory into segments starting and ending at the boundary states (A and B) [2]. If these segments are used, then $Z_{C,1}(q, \Delta t) = N_{AB}$ for all q and Δt, which is used as a committor validation test, as we did in the present study.

Finally, as the non-normalized histogram scales with Δt like $Z_H \sim \Delta t^{-1}$, the fact that $Z_{C,1}(q, \Delta t)$ is independent of Δt together with Eq. 12 implies that $D(q, \Delta t)$ is also independent of Δt, in the same way that is expected for normal Markovian diffusion. Nevertheless, as the condition of a $D(r, \Delta t)$ (computed via Eq.12) independent of Δt is a necessary (but not sufficient) condition for the projection over r to be Markovian, it can still be that the projection over q is not strictly Markovian [6]. Even so, the diffusive model along it can be used to compute exactly important kinetic properties, the equilibrium flux J, the mean first passage times and the mean transition path times [6].

Free-energy profiles with unitary diffusion coefficient

In the main text we presented the free-energy profiles over a rescaled coordinate $\tilde{q}$ where the diffusion coefficient is unitary. Such transformation can always be performed for one-dimensional diffusive models [10, 11]. This is a consequence of the fact that $Z_{C,0}$ is invariant under an arbitrary continuous invertible transformation of the coordinate space [10]. Particularly, we consider the transformation $\tilde{q} = \tilde{q}(q)$ with q being the committor and $\tilde{q}$ is the rescaled coordinate where $D(\tilde{q}, \Delta t) = 1$ and one has $Z_{C,0}(q, \Delta t) = Z_{C,0}(\tilde{q}, \Delta t)$. On the other hand, Z_H is not invariant under such transformation since it satisfies [10] $Z_H(q, \Delta t)\mathrm{d}q = Z_H(\tilde{q}, \Delta t)\mathrm{d}\tilde{q}$. Using the invariance of $Z_{C,0}$ and relations like Eq. 12 between $Z_{C,0}$, $Z_{C,1}$ and Z_H, see Ref. [9], one can find

$$\tilde{q}(q) = \sqrt{\Delta t} \int_0^q \sqrt{\frac{Z_H(q', \Delta t)}{Z_{C,1}(q', \Delta t)}} \mathrm{d}q', \tag{14}$$

from which one easily sees that, because $Z_H(q, \Delta t) \sim \Delta t^{-1}$ and $Z_{C,1}(q, \Delta t)$ is in fact independent of Δt, the transformation is also independent of Δt for an optimal coordinate q. On the other hand, for suboptimal coordinates, like the n-based $q(n)$ RC defined in the main text, such transformation will depend on Δt as it is also $D(q(n), \Delta t)$. In any case, we performed the transformation on the smallest timescale $\Delta t = \Delta t_0 = 400$MCs as our aim was to compare the coordinates with the highest temporal resolution available.

Kinetic properties from diffusive models

Here we briefly review how kinetic properties related to the original complex multidimensional dynamics can be calculated from one-dimensional diffusive models, namely the equilibrium flux J, the mean first passage times (MFPT) and the mean transition path times (MTPT).

The equilibrium flux J is given by the number of transitions per unit time $N_{AB}/\mathcal{N}\Delta t_0$ with $\mathcal{N}$ being the trajectory length sampled with Δt_0, so that N_{AB} is the quantity representative of this kinetic property. From the diffusive model along the committor q, J can be computed exactly by [12]

$$J = D(q)p_{\mathrm{eq}}(q) = D(q)Z_H(q)/\mathcal{N}, \tag{15}$$

where $p_{\mathrm{eq}}(q) = Z_H(q)/\mathcal{N} = e^{-\beta F(q)}/\mathcal{N}$ is just the equilibrium distribution of q and $\beta = 1/k_BT$. And, from Eq 12, this can be related to $Z_{C,1}(q)$ as

$$J = \frac{Z_{C,1}(q)}{\mathcal{N}\Delta t_0} = \frac{N_{AB}}{\mathcal{N}\Delta t_0}. \tag{16}$$

From the above result one sees that the criteria $Z_{C,1}(q) = N_{AB}$ independent of q and Δt is related to the fact that the diffusive model along q provides the exact expression for J at any timescale. Moreover, as the integral of $Z_{C,1}$ is related to the total squared displacement (TSD) by [2]

$$\frac{\text{TSD}}{2} = \int_0^1 Z_{C,1}(q)\mathrm{d}q = N_{AB}, \tag{17}$$

so the number of transitions N_{AB} can be estimated directly as half of the TSD of the committor.

The MFPT from aggregated (A) to diluted (B) states $\tau_{A\to B}$ can be computed exactly by [7, 13]

$$\tau_{A\to B} = \int_0^1 e^{\beta F(q')}\frac{\mathrm{d}q'}{D(q')}\int_0^{q'} e^{-\beta F(q'')}\mathrm{d}q'' \tag{18}$$

and that can be rewritten as [6, 7, 14]

$$\tau_{A\to B} = \langle 1-q\rangle/J \tag{19}$$

where $\langle ...\rangle$ denotes equilibrium averages. Both equations led to similar results for the aggregating transitions occurring in our model system, that is, numerically integrating Eq. 18 or taking directly the mean of $(1-q)$ and using N_{AB} (J) computed from the TSD in Eq. 19. Similarly, the mean first passage time from diluted to aggregated states $\tau_{B\to A}$ can be computed as [7]

$$\tau_{B\to A} = \int_0^1 e^{\beta F(q')}\frac{\mathrm{d}q'}{D(q')}\int_{q'}^1 e^{-\beta F(q'')}\mathrm{d}q'' = \langle q\rangle/J. \tag{20}$$

Finally, the MTPT $\hat{\tau}$ between aggregated and diluted states, which is equal in both directions [15], can be computed exactly as [6, 16]

$$\hat{\tau} = \frac{\int_0^1 e^{-\beta F(q')}\mathrm{d}q'\left(\int_0^{q'} e^{\beta F(q'')}\frac{\mathrm{d}q''}{D(q'')}\right)\left(\int_{q'}^1 e^{\beta F(q'')}\frac{\mathrm{d}q''}{D(q'')}\right)}{\int_0^1 e^{\beta F(q')}\frac{\mathrm{d}q'}{D(q')}} = \langle q(1-q)\rangle/J. \tag{21}$$

In this section, we dropped the Δt dependence of all quantities, but it is worth noting that for all results showed in the main text the computations were performed with $\Delta t = \Delta t_0 = 400$MCs, so the results of the diffusive models were evaluated with the highest temporal resolution available.

Additional results

Here we show some additional results for larger lattice systems, as well as one result for a (3D) Lennard-Jones system.

Lattice systems

In Table SI we show $\tau_{B\to A}$ calculated via Eq. 20 and directly from the RC time series for both $q(n)$ and q, as well as the number of transitions N_{AB} calculated from $Z_{C,1}$ via Eq. 17 and direct from the time series also for both RCs, for different system sizes. We consider isotropic ($\xi = 1$) systems of size $L = 200$ containing $N = 400$ molecules (same as in the main text), $L = 260$ containing $N = 676$ molecules and $L = 320$ containing $N = 1024$, so that the concentration is fixed at $\rho = N/L^2 = 10^{-2}$. We note that the discrepancies observed for the diffusive models along the n-based coordinate are persistent with increasing system size, and that the diffusive model over our proposed optimized coordinate q can be used to describe the kinetics of all system sizes considered. For the system with $N = 400$ molecules six simulations of length 10^8 MCs were considered while for the system with $N = 676$ we considered four simulations of the same length, and for the system with $N = 1024$ we considered four simulations but with doubled length. We note that for the largest system we used a slightly different temporal resolution $\Delta t_0' = 2.5\Delta t_0 = 10^3$MCs due to hardware limitations.

Finally, in Table SII we show other two characteristic times for the same systems considered in the main text, the MFPT from aggregated to diluted states $\tau_{A\to B}$ and the MTPT $\hat{\tau}$. Again, we show these quantities computed from the diffusive model to see how they compare with the results obtained directly from the time series, for both $q(n)$ and q RCs. Similarly to the quantities presented in the main text, the diffusive models over $q(n)$ result in kinetics that are about three to four times faster, while for the proposed optimum coordinate q the estimated values are virtually the same as the values measured from the time series. All data in Table SII were obtained from six simulations of length 10^8 MCs. In both tables, we also list the values of the inverse temperature $(T^*)^{-1}$ used in the Metropolis MC simulations for each system, in units where k_B and ψ_w are unitary.

TABLE SI. Estimates for the MFPT $\tau_{B\to A}$ and the number of transitions N_{AB} obtained from both the n-based RC $q(n)$ and the commitor q for isotropic lattice systems ($\xi = 1$) with different numbers of molecules N with the same concentration. All times are given in units of $10^3 \times \Delta t_0$.

	$N = 400$	$N = 676$	$N = 1024$
$(T^*)^{-1}$	2.73087	2.69033	2.66521
$\tau_{B\to A}$ from Eq. 20 with $q(n)$	1.8	3.1	9.9
$\tau_{B\to A}$ from time series of $q(n)$	4.7	8.7	23
N_{AB} from $Z_{C,1}(q(n), \Delta t_0)$	336	108	63
N_{AB} from time series of $q(n)$	122	30	24
$\tau_{B\to A}$ from Eq. 20 with q	5.4	13	31
$\tau_{B\to A}$ from time series of q	5.3	11	26
N_{AB} from $Z_{C,1}(q, \Delta t_0)$	113	29	21
N_{AB} from time series of q	112	27	24

TABLE SII. Estimates for the MFPT $\tau_{A\to B}$ and the MTPT $\hat{\tau}$ obtained from both the n-based RC $q(n)$ and the commitor q for lattice systems with different anisotropies ξ. All times are given in units of $10^3 \times \Delta t_0$.

	$\xi = 1$	$\xi = 3$	$\xi = 5$	$\xi = 7$
$(T^*)^{-1}$	2.73087	1.61744	1.13764	0.89222
$\tau_{A\to B}$ from Eq. 18 with $q(n)$	2.9	5.9	6.1	7.6
$\tau_{A\to B}$ from time series of $q(n)$	7.6	19	26	32
$\hat{\tau}$ from Eq. 21 with $q(n)$	0.38	0.6	0.7	1.5
$\hat{\tau}$ from time series of $q(n)$	0.94	1.5	2.6	6.1
$\tau_{A\to B}$ from Eq. 18 with q	7.9	18	25	31
$\tau_{A\to B}$ from time series of q	8.0	19	27	30
$\hat{\tau}$ from Eq. 21 with q	1.2	1.9	2.9	6.8
$\hat{\tau}$ from time series of q	1.2	1.9	2.9	8.1

Lennard-Jones system

In order to demonstrate the generality of our approach we also consider a Lennard-Jones (LJ) system in three spatial dimensions. In addition to being a 3D system, the dynamics here were simulated by means of the overdamped Langevin equation, in contrast to the Monte Carlo scheme used for the lattice system. We consider a system with $N = 100$ particles contained in a cubic box of $V = L^3$, in a way that the concentration is $\rho = N/V = 10^{-2}$ (similar to the lattice case) in length units where the diameters of each particle is unitary. The interaction between a pair of particles i and j separated by a distance d_{ij} is given the LJ potential

$$V_{ij}(d_{ij}) = 4\epsilon \left[\left(\frac{\sigma}{d_{ij}} \right)^{2\alpha} - \left(\frac{\sigma}{d_{ij}} \right)^{\alpha} \right] \tag{22}$$

where σ is the diameter of each particle (set to unity), ϵ defines the depth of the potential and energy scale (we set $\epsilon = 5$ arbitrary units of energy). Here we used the generalization of the LJ potential with $\alpha = 10$ because we were following Ref. [17] that were trying to describe some colloidal experimental data. Choices of α different from the standard $\alpha = 6$ are useful for describing colloidal systems since it allows modifying the range of the interaction but no drastic differences near the coexistence aggregation line are expected for the value used here [18]. Indeed we find that at a temperature $T = 1.92$ (in units were k_B is unitary) the system undergoes a first-order phase transition characterized by bimodal distributions of the order parameters as we show next. The overdamped Langevin equations for each particle were integrated using Euler's method [19] with a time discretization of $\delta t = 0.01$ and a fixed diffusion coefficient $D_0 = 0.005$ (on physical space) similar to Ref. [17]. Configurations were saved at every 8×10^3 integration steps so that $\Delta t_0 = 8 \times 10^3 \delta t$. With five long equilibrium simulations (each with 6×10^9 integration steps) we observed $N_{AB} = 24$ transitions between diluted and aggregated states.

In Fig. S3 we show the analogue of Fig 1 of the main article, but now for the LJ system. We again compare the n-based coordinate $q(n)$ with our proposed optimum committor q. In this case the n coordinate is computed assuming that particles closer than a certain threshold (1.5σ) belong to the same cluster. Then, n is just the number of particles in the largest cluster, as in the lattice system case. The conclusions are very similar to what we discussed for the lattice system in the main text. $q(n)$ fails to pass the $Z_{C,1}$ validation test since $-\ln Z_{C,1}(q, \Delta t)$ changes considerably from -4.8 to -3.2 as Δt increases from Δt_0 to $2^{15}\Delta t_0$ as can be seen in Fig. S3 (a). Conversely, $Z_{C,1}(q, \Delta t)$ only fluctuates between the expected value of $-\ln Z_{C,1}(q, \Delta t) \approx -\ln N_{AB} \approx -3.2$ for all Δt up to the statistical uncertainty $1/\sqrt{2N_{AB}} \approx 0.15$, as can be seen in Fig. S3 (b). Also similar to the lattice case, the free-energy

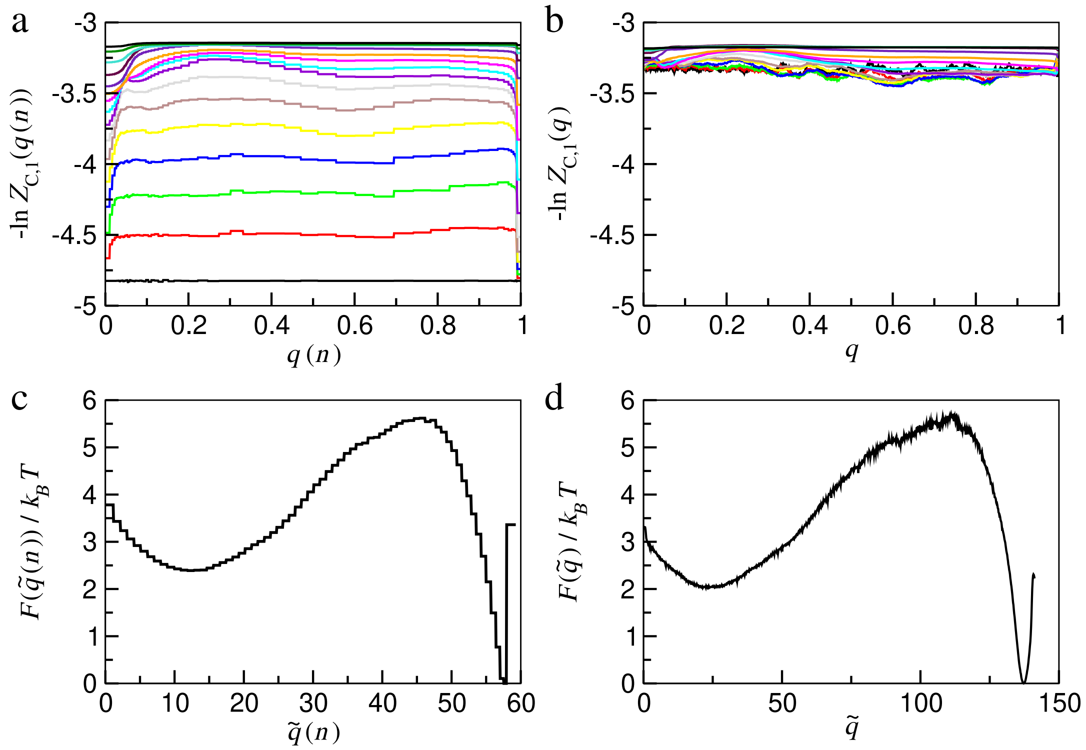

FIG. S3. Functions $-\ln Z_{C,1}(r, \Delta t)$ and free-energy profiles $F(r)/k_BT$ for the n-based RC $q(n)$ and the optimized committor q. Panels (a) and (b) show the $Z_{C,1}$-based committor validation tests for both RCs, where different colors correspond to different $\Delta t = 2^i \Delta t_0$, with $i = 0, 1, 2, ..., 15$ (from the bottom to the top). In panels (c) and (d) the profiles are presented as a function of the rescaled RCs where $D(r, \Delta t_0) = 1$.

TABLE SIII. Estimates for the number of transitions N_{AB}, MTPT $\hat{\tau}$ and MFPTs, $\tau_{A\to B}$ and $\tau_{B\to A}$, obtained from both the n-based RC $q(n)$ and the commitor q for the Lennard-Jones system. All times are given in units of $10^4 \times \Delta t_0$.

N_{AB} from $Z_{C,1}(q(n), \Delta t_0)$	125	$\tau_{A\to B}$ from Eq. 18 with $q(n)$	1.6
N_{AB} from time series of $q(n)$	23	$\tau_{A\to B}$ from time series of $q(n)$	10
$\hat{\tau}$ from Eq. 21 with $q(n)$	0.11	$\tau_{B\to A}$ form Eq. 20 with $q(n)$	1.4
$\hat{\tau}$ from time series of $q(n)$	0.41	$\tau_{B\to A}$ from time series of $q(n)$	7.0
N_{AB} from $Z_{C,1}(q, \Delta t_0)$	28	$\tau_{A\to B}$ from Eq. 18 with q	7.1
N_{AB} from time series of q	24	$\tau_{A\to B}$ from time series of q	9.9
$\hat{\tau}$ from Eq. 21 with q	0.34	$\tau_{B\to A}$ form Eq. 20 with q	6.3
$\hat{\tau}$ from time series of q	0.37	$\tau_{B\to A}$ from time series of q	6.8

profiles for the rescaled coordinates where the diffusion coefficient is unitary presents a similar free-energy barriers. The difference is again mostly due the ranges spanned between aggregated A and diluted B states, with the $q(n)$ RC exhibiting a much narrower range, which leads to faster kinetics as discussed in the main text. To corroborate that we include in Table SIII the number of transitions N_{AB}, MTPT $\hat{\tau}$ and MFPT's $\tau_{A\to B}$ and $\tau_{B\to A}$ obtained directly from the simulated trajectories and from the diffusive models along both coordinates. The $q(n)$ RC gives kinetics about five times faster the correct ones, while our proposed q coordinate describes all quantities with fair accuracy. The deviations are a little bit bigger than the lattice system case as should be expected due to the worse statistics of the present case with only $N_{AB} = 24$, in close relation to the fact that $Z_{C,1}(q, \Delta t)$ varies a bit more with Δt here.

References

[1] N. S. M. Herringer, S. Dasetty, D. Gandhi, J. Lee, and A. L. Ferguson, J. Chem. Theory Comput. **20**, 178 (2024).

[2] S. V. Krivov, J. Chem. Theory Comput. **9**, 135 (2013).

[3] P. V. Banushkina and S. V. Krivov, J. Chem. Phys. **143**, 184108 (2015).

[4] S. V. Krivov, J. Chem. Theory Comput. **14**, 3418 (2018).

[5] S. V. Krivov, J. Chem. Theory Comput. **17**, 5466 (2021).

[6] P. V. Banushkina and S. V. Krivov, WIREs Comput. Mol. Sci. **6**, 748 (2016).

[7] A. M. Berezhkovskii and A. Szabo, J. Phys. Chem. **150**, 054106 (2019).

[8] Y. Chen, J. Hoskins, Y. Khoo, and M. Lindsey, J. Comput. Phys. **472**, 111646 (2023).

[9] S. V. Krivov, J. Phys. Chem. B **115**, 11382 (2011).

[10] S. V. Krivov and M. Karplus, Proc. Natl. Acad. Sci. U.S.A. **105**, 13841 (2008).

[11] Y. M. Rhee and V. S. Pande, J. Chem. Phys. **109**, 6780 (2005).

[12] A. M. Berezhkovskii and A. Szabo, J. Phys. Chem. B **117**, 13115 (2013).

[13] E. Vanden-Eijnden, M. Venturoli, G. Ciccotti, and R. Elber, J. Chem. Phys. **129**, 174102 (2008).

[14] E. Vanden-Eijnden and M. Venturoli, J. Chem. Phys. **131**, 044120 (2009).

[15] A. M. Berezhkovskii, M. A. Pustovoit, and S. M. Bezrukov, J. Chem. Phys. **119**, 3943 (2003).

[16] A. M. Berezhkovskii, L. Dagdug, and S. M. Bezrukov, J. Phys. Chem. B **121**, 5455 (2017).

[17] F. Sciortino, P. Tartaglia, and E. Zaccarelli, J. Phys. Chem. B **109**, 21942 (2005).

[18] G. A. Vliegenthart, J. F. M. Lodge, and H. N. W. Lekkerkerker, Physica A **263**, 378 (1999).

[19] T. N. Azevedo and L. G. Rizzi, J. Phys.: Conf. Ser. **1483**, 012001 (2020).
